# The Oncogenic Role of miR-BART19-3p in Epstein-Barr Virus-Associated Diseases

**DOI:** 10.1155/2020/5217039

**Published:** 2020-07-02

**Authors:** Qingxun Zhang, Donghua Luo, Zhengde Xie, Hongxuan He, Ziyuan Duan

**Affiliations:** ^1^Institute of Zoology, Chinese Academy of Sciences, Beijing, China; ^2^Genetic Research Center, Institute of Genetics and Developmental Biology, Chinese Academy of Sciences, Beijing, China; ^3^University of Chinese Academy of Sciences, Beijing, China; ^4^Sun Yat-sen University Cancer Center, State Key Laboratory of Oncology in South China, Collaborative Innovation Center for Cancer Medicine, Guangdong Key Laboratory of Nasopharyngeal Carcinoma Diagnosis and Therapy, Guangzhou, China; ^5^Beijing Pediatric Research Institute, Beijing Children's Hospital, Capital Medical University, China

## Abstract

Accumulating evidence so far has shown that EBV's miRNAs have been found to be involved in cancer progression. However, the comprehensive EBV miRNA expression profiles and their biological significance in EBV-associated diseases are not well documented. A comprehensive profiling of EBV-encoded miRNAs expressed in CAEBV, EBV-HLH, and nasopharyngeal carcinoma (NPC) patients was constructed, and the results showed that miR-BART19-3p was upregulated in all these diseases. Ectopic expression of miR-BART19-3p induced EBV-negative cell proliferation and suppressed cell apoptosis. Molecularly, adenomatous polyposis coli (APC) was identified to be a direct target of miR-BART19-3p, and APC mRNA expression was inversely correlated with miR-BART19-3p in CAEBV samples. Our results demonstrated that miR-BART19-3p contributes to the tumorigenesis of EBV-associated diseases and may be a potential therapeutic target.

## 1. Introduction

Epstein-Barr virus (EBV), one of eight human herpesviruses, infects over 95% of the population worldwide. EBV persists lifelong as a latent infection in the B lymphoid system and maintains a finely balanced relationship with humans. Once the delicate EBV-host balance is broken, EBV can display its pathogenic potential [1]. Primary infection with EBV in adolescence frequently results in acute infectious mononucleosis (IM) [2]. In some cases, EBV can infect T cells and NK cells and induce chronic active EBV infection (CAEBV) [3], EBV-associated hemophagocytic lymphohistiocytosis (EBV-HLH) [4], and EBV-associated T/NK cell lymphoproliferative diseases [5]. CAEBV is classified as a lymphoproliferative disorder in the 2016 World Health Organization lymphoma classification [6]. The main symptoms of this disease are prolonged or relapsing IM-like symptoms and often progress to life-threatening hemophagocytic syndrome. CAEBV usually occurs following primary EBV infection and is mainly characterized by clonal proliferation of EBV-infected T/NK cells and inflammatory cytokine production [7]. But the mechanism by which EBV induces proliferation of T/NK cells and cytokines has not been elucidated. In addition, EBV is etiologically linked to several human malignancies, including Burkitt's lymphoma (BL), undifferentiated nasopharyngeal carcinoma (NPC), and EBV-associated gastric carcinoma (EBVa GC) [1].

Apart from latent proteins (EBNAs, LMP1, and LMP2A) and EBV-encoded RNAs (EBERs), EBV also expresses 44 mature microRNAs including BamHI fragment H rightward open reading frame 1 (BHRF1) miRNAs and BamHI A rightward transcript (BART) miRNAs [8]. These miRNAs are differentially expressed in different cell types and latencies, which have been extensively studied in lymphoma and carcinoma [9–11]. Emerging findings suggest that EBV's miRNAs are involved in regulating innate and adaptive immune responses, cell proliferation and apoptosis, and tumor metastasis [12–14]. EBV-derived noncoding RNAs especially miRNAs can also transfer through exosome and regulate the function for the tumorigenesis [15]. Previous study showed that several Wnt inhibitory genes, including Wnt inhibitory factor 1 (WIF1), Nemo-like kinase (NLK), and adenomatous polyposis coli (APC), were downregulated by EBV miR-BART19-3p [16], which might increase cell proliferation. There is a developing hypothesis and increasing evidence that CAEBV should be considered a neoplastic disease. Their results indicated that CAEBV partly originates from an EBV-infected lymphoid progenitor that acquires DDX3X and other mutations. For another, the EBV genome in CAEBV patients harboured frequent intragenic deletions in two immediate early genes (BZLF1 and BRLF1) and BART miRNA-encoding region [17]. Despite recent advances, a systemic investigation on the expression profile of EBV miRNAs expressed in CAEBV and EBV-HLH, as well their biological significance in EBV-associated diseases, is needed to understand.

In the present study, we constructed a comprehensive profiling of 44 known EBV miRNAs in clinical samples from CAEBV, EBV-HLH, and NPC patients and identified several viral miRNAs that were frequently highly expressed. Furthermore, we tried to explore the interactions between miR-BART19-3p, one of the highly expressed EBV miRNAs in latency II, and host tumor cells. Interestingly, we demonstrated that miR-BART19-3p induced cell growth and suppressed apoptosis by targeting adenomatous polyposis coli (APC), which might help dissect how EBV miRNAs contribute to the development of EBV-associated diseases.

## 2. Materials and Methods

### 2.1. Cell Lines

Four EBV-positive cell lines were used: the EBV-positive Burkitt lymphoma cell line Akata-Bx1 (type I latency), EBV-positive gastric carcinoma cell line AGS-EBV (modified latency I), EBV-positive nasopharyngeal carcinoma-derived cell line C666-1 (type II latency), and EBV-immortalized lymphoblastoid cell line B95-8 (type III latency). CNE2 was non-EBV nasopharyngeal carcinoma cell line. T-ALL cell line Jurkat was an EBV-negative cell line. All cell lines were preserved in our laboratory and were routinely cultivated in 1640 medium with 10% heat-inactivated fetal bovine serum (Gibco), 100 U/ml penicillin, and 100 *μ*g/ml streptomycin (Invitrogen, CA).

### 2.2. Clinical Patients

50 subjects including 20 chronic active EBV infection (CAEBV) patients, 20 EBV-associated hemophagocytic lymphohistiocytosis (EBV-HLH) patients, and 10 healthy children were enrolled in this study at Beijing Children's Hospital between April 2016 and June 2017. Peripheral blood sample with EDTA was collected from all 50 subjects at the time of diagnosis (Supplementary Table 1). In addition, 14 NPC tumor tissues, 4 adjacent tissues, and 3 nasopharyngeal mucosal chronic inflammation tissues were obtained from Sun Yat-sen University Cancer Center in May 2018 (Supplementary Table 2). The study was approved by the Ethics Committee of the Institute of Zoology, and the protocol follows the institutional guidelines for human welfare.

### 2.3. RNA Extraction, Reverse Transcription, and Relative Quantification of miRNAs and mRNAs

Total RNAs, including miRNAs, were purified and enriched with the miRcute miRNA Isolation Kit (TIANGEN, Beijing, China) following the manufacturer's standard protocol. EBV-encoded miRNAs were polyadenylated by poly (A) polymerase, and the miRcute miRNA First-Strand cDNA Synthesis Kit was used to generate cDNA. Real-time PCR was performed using the miRcute miRNA qPCR detection kit (TIANGEN) according to the manufacturer's protocol. Forward primers (Supplementary Table 3) were designed on the basis of the EBV miRNA mature sequences obtained from miRBase (http://www.mirbase.org), and the reverse primer was provided by the kit. The mRNA levels were quantified using a real-time PCR system with the SYBR Green qPCR kit (Takara, Japan). Specific primers for APC and *β*-actin used were as follows: APC, 5′-AAAGTGAGCAGCTACCACGG-3′ (forward) and 5′-CCTGGAGTGATCTGTTAGTCG-3′ (reverse); *β*-actin, 5′-GTGGGCCGCTCTAGGCACCA-3′ (forward) and 5′-CGGTTGGCCTTAGGGTTCAGGGG-3′ (reverse). U6 snRNA and *β*-actin were used for normalizing the expression of miRNAs and mRNAs. The fold changes were calculated by using the 2-*ΔΔ*Ct method.

### 2.4. Transient Transfection

MicrONTM miR-BART19-3p mimic (5′-UUUUGUUUGCUUGGGAAUGCU-3′), micrONTM miR-BART19-3p-mutant (5′-UAUGCGAUGCUUGGGAAUGCU-3′), micrOFFTM miR-BART19-3p inhibitor (5′-AGCAUUCCCAAGCAAACAAAA-3′), and its appropriate negative control were purchased from RiboBio (Guangzhou, China). siRNA-NC and siRNA-APC were purchased from GenePharma (Shanghai, China). siR-APC, miR-BART19-3p mimic, miR-BART19-3p-mutant, and miR-BART19-3p inhibitor were transfected into target cells at a final concentration of 40 nmol/l, 50 nmol/l, 50 nmol/l, and 100 nmol/l using Lipofectamine 3000 (Invitrogen) according to the manufacturer's instructions.

### 2.5. Methylthiazol Tetrazolium (MTT) Assay and EdU Incorporation Assay

Cell proliferation was measured by using MTT assay. After transfection, 3 × 10^3^ per well were seeded in 96-well plates, and viability was assessed in six replicates at 24, 48 h, and 72 h. 20 *μ*l MTT was added, and the mixture was incubated for 4 h at 37°C. Subsequently, 150 *μ*l dimethylsulfoxide (Sigma-Aldrich, St. Louis, MO, USA) was added to each well, followed by thorough mixing for 10 min. The absorbance was measured using an ELISA microplate reader (680; Bio-Rad, Hercules, CA, USA) at a wavelength of 490 nm. EdU incorporation assays were performed using Click-iT EdU Imaging Kit (US EVERBRIGHT INC.) according to the manufacturer's instructions.

### 2.6. Flow Cytometry

Target cells were plated onto 6-well plates and transfected with EBV miR-BART19-3p mimic or negative control oligonucleotide, respectively. Cell apoptosis analysis was performed using the Annexin V-FITC Apoptosis Analysis Kit (Beyotime Institute of Biotechnology, Shanghai, China) according to the recommended protocol. Events were recorded from samples using a FACSCalibur Flow Cytometer (BD Biosciences, USA), and the data were analyzed using ModFit software (Verity Software House, Topsham, ME).

### 2.7. Luciferase Reporter Assay

The miR-BART19-3p binding site-containing 3′UTR fragment of APC (about 1208 bp) was amplified and cloned into the modified pGL3-luciferase vector. The CNE2 cells were transfected with 0.1 *μ*g of APC-3′UTR luciferase vector and 50 nM of the miR-BART19-3p mimic, miR-BART19-3p-mut, or miR-control together with a *β*-galactosidase plasmid using Lipofectamine 3000 according to the manufacturer's instructions. Luciferase activities were measured with a luciferase assay kit (Promega) at 48 h after transfections.

### 2.8. Western Blotting

Whole cell lysates were extracted 48 h post transfection using RIPA lysis buffer. Western blotting was performed as previously described [18]. The commercial antibodies used in this study were anti-APC (ImmunoWay catalog no. YT0258) and anti-*β*-actin (CW0096M, CWBIO). HRP-conjugated secondary antibodies were from Jackson ImmunoResearch.

### 2.9. Statistical Analysis

Experimental data were analyzed using SPSS 25.0 (SPSS) and GraphPad Prism 7 (GraphPad) and presented as the mean ± SD of three independent experiments. Student's *t*-test was used for the differences between groups. The associations between miR-BART19-3p expression and the APC level in clinical samples were calculated by Spearman's correlation coefficient. *p* values < 0.05 (∗*p* < 0.05, ∗∗*p* < 0.01, and ∗∗∗*p* < 0.001) were considered statistically significant.

## 3. Results

### 3.1. Expression Profile Analysis Showed That miR-BART19-3p Was Upregulated in EBV-Associated Diseases

**Figure 1:**
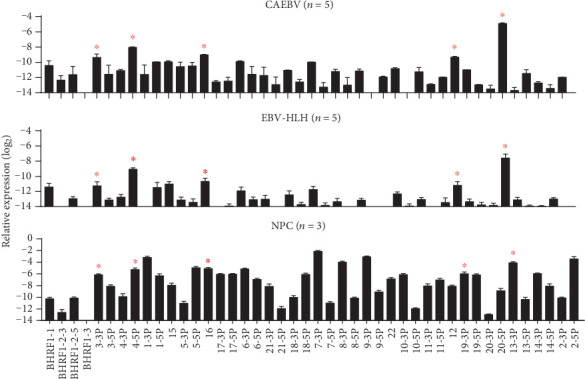
Comprehensive and quantitative profiling of EBV-miRNAs in CAEBV, EBV-HLH, and NPC patients using poly (A)-tailed real-time quantitative polymerase chain reaction.

**Figure 2:**
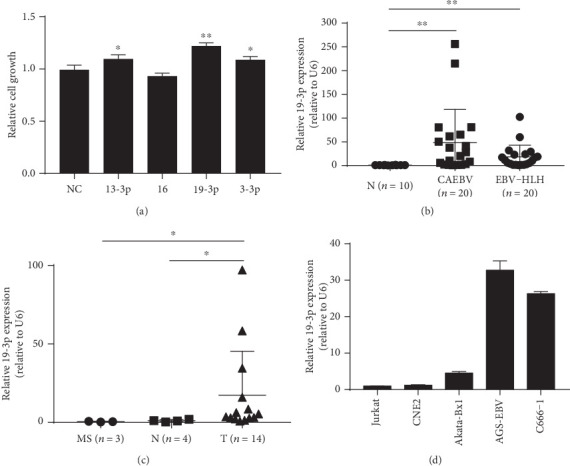
miR-BART19-3p was significantly upregulated in EBV-associated diseases. (a) Effects of five highly expressed miRNAs on cell growth. (b) Relative expression of miR-BART19-3p in a cohort of 20 CAEBV and 20 EBV-HLH samples compared with healthy control. (c) Relative expression of miR-BART19-3p in NPC tumor tissues, 4 adjacent tissues, and 3 nasopharyngeal mucosal chronic inflammation tissues. (d) Relative expression of miR-BART19-3p in EBV-positive and EBV-negative cell lines. ∗*p* < 0.05, ∗∗*p* < 0.01, Student's *t*-test, *n* = 3 independent experiments.

In the present study, we mainly used real-time RT-PCR to gain insight into the comparable expression pattern of forty-four known EBV miRNA transcriptomes of EBV-associated diseases (Figure 1). The overexpressed EBV miRNA was predominantly in the BART region, whereas the expression of the BHRF1 family was nearly absent. Previous studies have shown that the expression of the BHRF1 cluster is latency III dependent [9]. Notably, the 13-3p, 4-5p, 16, 19-3p, 3-3p, 1-5p, 7-3p, 6-3p, and 15 miRNAs from the BART region showed significant fold changes in CAEBV, EBV-HLH, and NPC patients. Highly expressed miRNAs (miR-BART13-3p, 16, 19-3p, and 3-3p) were synthesized and transfected in CNE2 cells to investigate the effects of BART miRNAs on cell growth. Among these miRNAs, we focused on miR-BART19-3p due to the effects on cell growth (Figure 2(a)). To investigate whether miR-BART19-3p expression was upregulated, we determined the expression of miR-BART19-3p in a cohort of 20 CAEBV and 20 EBV-HLH samples and healthy controls and also in 14 NPC tumor tissues and 4 adjacent tissues. A substantial higher level of miR-BART19-3p was observed in these samples than control (Figures 2(b) and 2(c)). Meanwhile, the expression of miR-BART19-3p in EBV-positive cell lines (Akata-BX1, AGS-EBV, and C666-1) was higher than that in negative cell lines (Jurkat and CNE2) (Figure 2(d)), which suggested that miR-BART19-3p might play a role in EBV-associated diseases.

### 3.2. miR-BART19-3p Induced Cell Growth and Suppressed Apoptosis

**Figure 3:**
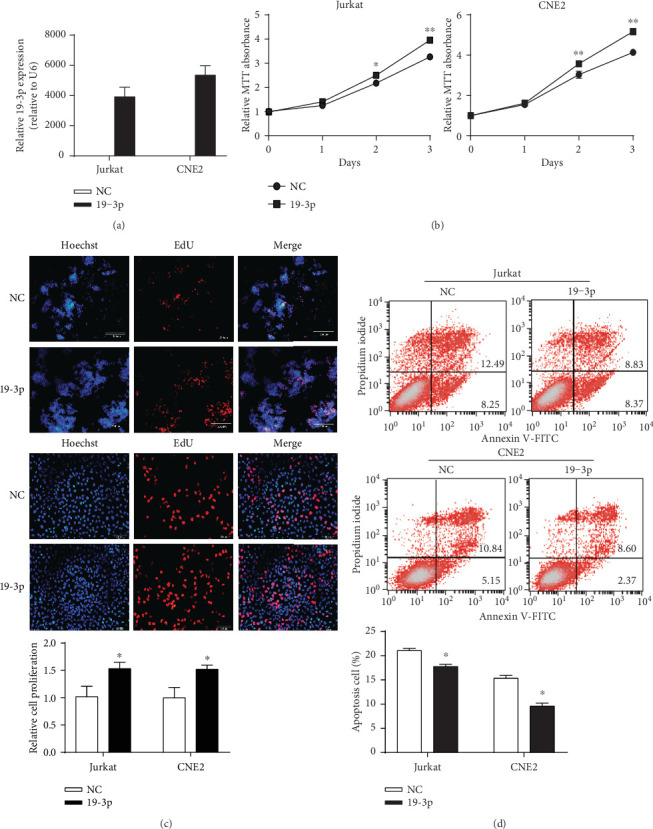
miR-BART19-3p overexpression promotes cell growth and inhibits apoptosis. (a) The expression of exogenous EBV-miR-BART19-3p was detected by q-PCR after transfection of miR-BART19-3p mimic at 48 h. (b) Cell proliferation ability was detected using MTT assay at 24, 48, and 72 hours. (c) EdU incorporation assay was measured after transfection of miR-BART19-3p mimic at 48 h. (d) Cell apoptosis rate was measured by flow cytometric analysis of Annexin V-FITC/PI staining after transfection of miR-BART19-3p mimic at 48 h. ∗*p* < 0.05, ∗∗*p* < 0.01, Student's *t*-test, *n* = 3 independent experiments.

**Figure 4:**
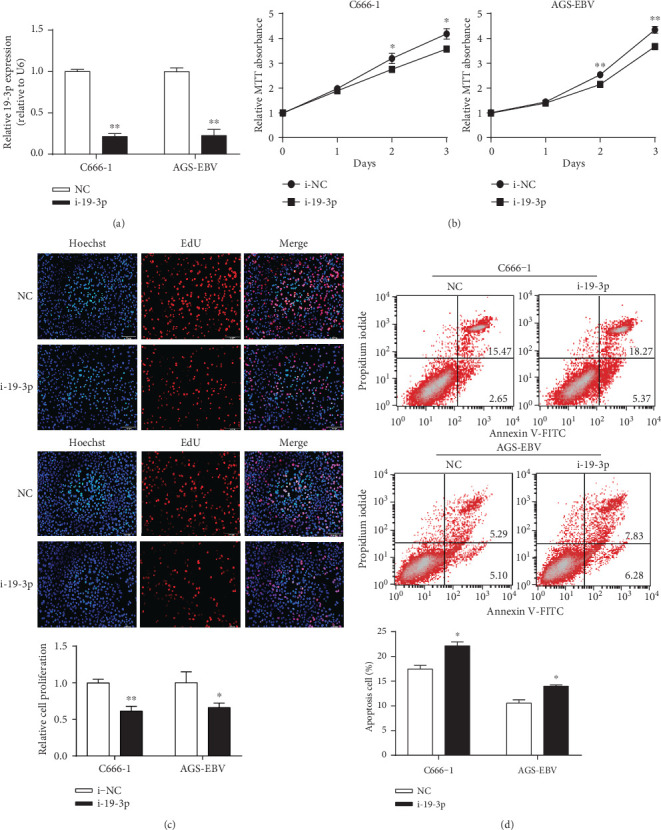
miR-BART19-3p knockdown inhibits growth and induces apoptosis. (a) The expression of EBV-miR-BART19-3p was detected by q-PCR after transfection of miR-BART19-3p inhibitor at 48 h. (b) Cell proliferation ability was detected using MTT assay at 48 hours. (c) EdU incorporation assay was measured after transfection of miR-BART19-3p inhibitor at 48 h. (d) Cell apoptosis rate was measured by flow cytometric analysis of Annexin V-FITC/PI staining after transfection of miR-BART19-3p inhibitor at 48 h. ∗*p* < 0.05, ∗∗*p* < 0.01, Student's *t*-test, *n* = 3 independent experiments.

To assess the potential role of miR-BART19-3p in EBV-associated diseases, we first evaluated the effects of miR-BART19-3p on cell growth and apoptosis using a gain-of-function approach. miR-BART19-3p mimic was transfected into EBV-negative cell lines (Jurkat and CNE2) to upregulate miR-BART19-3p level (Figure 3(a)). MTT assay suggested miR-BART19-3p overexpression promoted Jurkat and CNE2 cell proliferation compared with cells transfected with miR-NC at 48 h and 72 h (Figure 3(b)). EdU incorporation assay suggested miR-BART19-3p overexpression significantly increased EdU-positive cell number compared to NC (Figure 3(c)). Furthermore, apoptotic cells were detected using the Dead Cell Apoptosis Kit with Annexin V-FITC and PI 48 h after transfection with miRBART19-3p mimic or miR-NC. The ratio of the apoptotic cell population in CNE2 cells transfected with miR-BART19-3p was 10.97% while that in CNE2 cells transfected with the miR-NC was 15.99% (*p* < 0.05) (Figure 3(d)). Similar results were obtained in Jurkat cells (Figure 3(d)). To further confirm the above results, we next examined the effects of miR-BART19-3p loss of function on EBV-positive cell growth and apoptosis. Transfection of C666-1 and AGS-EBV with miR-BART19-3p inhibitor to inhibit endogenously expressed miR-BART19-3p (Figure 4(a)) resulted in reduced cell growth (Figures 4(b) and 4(c)) and increased apoptosis (Figure 4(d)).

### 3.3. APC Is a Target of EBV-miR-BART19-3p

**Figure 5:**
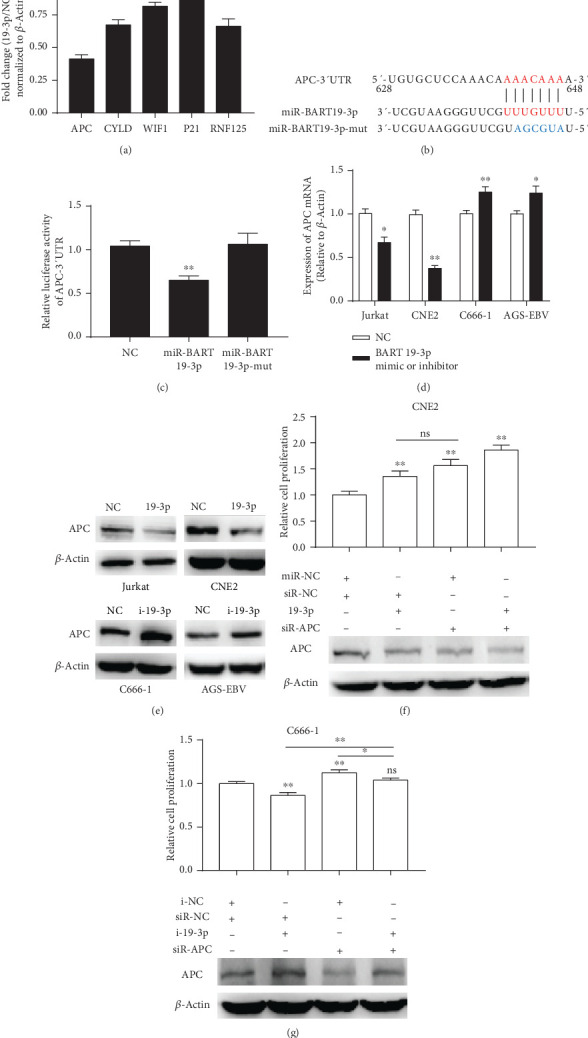
APC is the target of miR-BART19-3p. (a) q-PCR validation of selected genes downregulated in CNE2 cells expressing miR-BART19-3p. (b) Predicted miRNA binding sites within the 3′-UTR of APC mRNA. The seed sites and the mutation in seed sites of miR-BART19-3p were shown. (c) Luciferase activity was measured in CNE2 cells transfected with the APC 3′-UTR luciferase reporter vector and miR-BART19-3p mimic, miR-BART19-3p-mut, or relative miRNA control. (d) APC mRNA expression levels in EBV-positive and EBV-negative cells after miR-BART19-3p mimic or miR-BART19-3p inhibitor treatment. (e) Expression of APC protein is reduced by the miR-BART19-3p mimic and increased by the miR-BART19-3p inhibitor. (f) EdU incorporation assay was measured after transfection of miR-BART19-3p mimic and siR-APC at 48 h in CNE2. (g) EdU incorporation assay was measured after transfection of miR-BART19-3p inhibitor and siR-APC at 48 h in C666-1. ∗*p* < 0.05, ∗∗*p* < 0.01, Student's *t*-test, *n* = 3 independent experiments.

Next, we investigated the targets of miR-BART19-3p. Based on the bioinformatics analysis using TargetScan, TargetRank, and RNAhybrid, five genes (APC, CYLD, WIF1, p21, and RNF125) were predicted to be targeted by miR-BART19-3p. To validate the downregulation of these genes, miR-BART19-3p mimic was expressed in CNE2, and the mRNA levels were measured by q-PCR (Figure 5(a)). However, APC was decided to focus on because it was the most significantly downregulated gene. To validate the direct interaction of miR-BART19-3p with APC-3′-UTR, we performed a series of luciferase reporter assays with cotransfection of miR-BART19-3p mimic, miR-BART19-3p-mut, and a luciferase reporter plasmid (APC-3′UTR) into CNE2 cells. The luciferase activity of APC-3′UTR vectors was significantly attenuated by approximately 40%, when compared to miR-control, but exhibited no effect on the miR-BART19-3p-mut (Figures 5(b) and 5(c)). To illustrate the regulatory effect of BART19-3p on endogenous APC expression, EBV-negative cell lines (Jurkat and CNE2) were transfected with a miR-control or miR-BART19-3p mimic. The overexpression of BART19-3p was measured using q-PCR. In cells transfected with the miR-BART19-3p mimic, APC expression was significantly inhibited (Figures 5(d) and 5(e)) at the mRNA and protein levels when compared with miR-control. In contrast, transfection of miR-BART19-3p inhibitor into EBV-positive cell lines resulted in the upregulation of APC mRNA and protein levels (Figures 5(d) and 5(e)). To test the off-target effect of miR-BART19-3p on cell growth, we knocked down miR-BART19-3p and APC in CNE2 and C666-1 (Figures 5(f) and 5(g)). If they have similar phenotypes or reverse, the phenotype caused miR-BART19-3p knockdown in CNE2 and C666-1, respectively, suggesting miR-BART19-3p promotes proliferation by inhibiting APC. In fact, EdU incorporation assay suggested that knockdown of miR-BART19-3p and APC in CNE2 significantly increased the number of EdU-positive cells but with no significance of these two groups (Figure 5(f)). Meanwhile, the phenotype of miR-BART19-3p inhibitor was reversed by transfection of APC siRNA (Figure 5(g)). These results suggested miR-BART19-3p indeed promotes cell proliferation by inhibiting APC.

**Figure 6:**
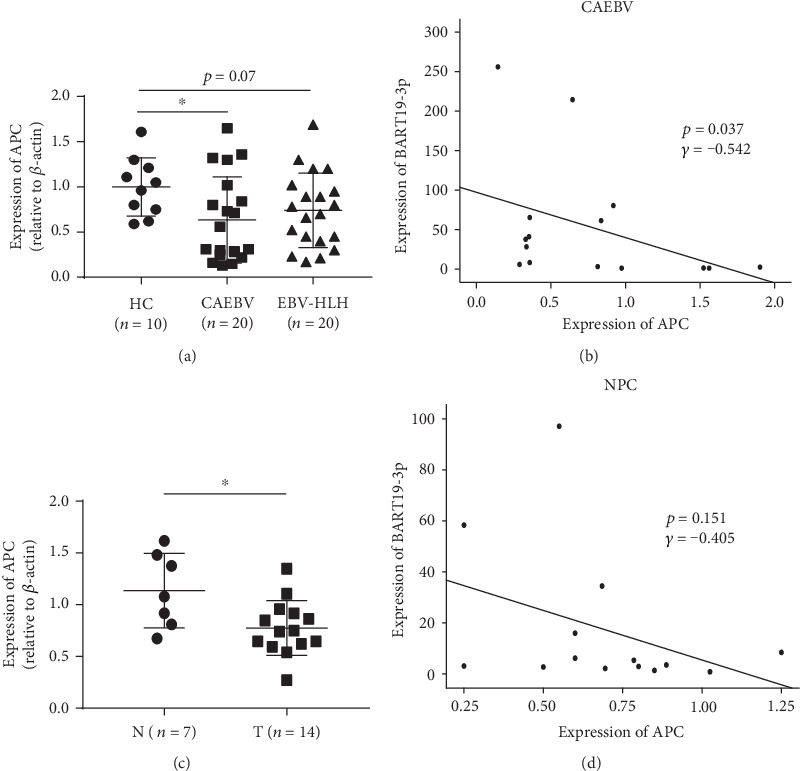
Expression of APC is often decreased and inversely correlated with miR-BART19-3p expression in CAEBV samples. (a) Relative APC expression levels in 20 CAEBV samples compared with healthy control. (b) Correlation analysis of miR-BART19-3p levels and APC levels in CAEBV patients. (c) Relative APC expression levels in NPC tumor tissues compared with control. (d) Correlation analysis of miR-BART19-3p levels and APC levels in NPC tumor tissues. ∗*p* < 0.05, Student's *t*-test.

The relationship between target genes and miR-BART19-3p expression was further investigated by directly comparing their mRNA expression levels in clinical samples. The APC expression levels were significantly lower in CAEBV and NPC patients, whereas no significant differences for EBV-HLH (Figures 6(a) and 6(c)). And only the levels of APC in CAEBV patients were negatively correlated with BART19-3p expression (Figures 6(b) and 6(d)). We conclude, therefore, that APC was the target of EBV miR-BART19-3p.

## 4. Discussion

To our knowledge, this is the first study to conduct comprehensive profiling and pattern analysis of 44 known EBV miRNAs in children with CAEBV. The comparison of EBV-miRNA pattern in CAEBV patients with EBV-HLH and NPC revealed that EBV could display modified type II latency in CAEBV. The integral profiling showed that the most abundant viral miRNAs in EBV latency II disease were miR-BART13-3p, followed by 4-5p, 16, 19-3p, 3-3p, 1-5p, 7-3p, 6-3p, and 15 in our study. Preliminary investigations showed that EBNA1, LMP2, and BARTs were abundantly detected in CAEBV patients, whereas LMP1 was detected less frequently and was expressed at lower levels [3]. Iwata et al. also reported that the results based on latent EBV genes demonstrated type II latency in CAEBV, which is consistent with our results.

Accumulating evidence so far has shown that EBV's miRNAs have been found to be involved in NPC, BL, HL, and EBVa GC progression [13, 19, 20]. The BH3-interacting domain death agonist (BID) has been reduced to the level of both mRNA and protein by EBV miR-BART4-5p in EBaGC cells [10]. And, miR-BART4-5p was also expressed greatly in CAEBV and EBV-HLH in our study. Therefore, we speculated that miR-BART4-5p might play a similar oncogenic role in different types of EBV-associated malignancy. Currently, some studies have identified EBV miRNAs as biomarkers for diagnosis and prognosis of EBV-associated diseases. Wong et al. evaluated twelve EBV miRNAs (miR-BART1-3p, 2-5p, 5, 6-5p, 6-3p, 7, 8, 9, 14, 17-5p, 18-5p, and 19-3p) in the serum of NPC patients but not in that of healthy individuals [19]. Levels of plasma EBV miRNAs (miR-BART2-5p, 13, and 15) were also dramatically increased in patients with CAEBV infection. And these miRNAs were potential biomarkers of disease severity or prognosis [20]. Recently, Okuno et al. found that the deletion of BART microRNA was found in some cases of CAEBV [17]. And in our study, the expression of several BART miRNAs was not detected, but we did not analyze the mutations and deletions of EBV genome. The functions of EBV genome deletions and mutations in EBV-associated diseases need more attention in the future.

In the present study, high expression of EBV-miR-BART19-3p was evident in CAEBV, EBV-HLH, and NPC patients and AGS-EBV and C666-1 cell lines, but not in Akata-Bx1 and B95-8 cell lines. miR-BART19-3p enhanced cell proliferation, and miR-BART13-3p and miR-BART3-3p had limited impact, while miR-BART16 reduced cell growth. Flow cytometry showed that miR-BART19-3p overexpression induced apoptosis resistance in EBV-negative cells. Conversely, miR-BART19-3p inhibition decreased resistance to apoptosis in EBV-positive cells. To explore the antiapoptotic role played by EBV miRNAs, we used q-PCR to quantify the levels of five tumor suppressor genes (APC, CYLD, WIF1, p21, and RNF125). As a result, only the APC expression levels were significantly lower in EBV-infected patients. Mechanism analysis revealed that APC was the target of miR-BART19-3p. Meanwhile, miR-BART19-3p levels in 20 CAEBV samples were significantly correlated inversely with APC expression levels. However, the results of NPC patients were not significant, which might be due to a small sample size. More samples were required to assess the correlation between the expression of BART19-3p and APC in NPC tumors in the future.

Aberrant activation of the Wnt/*β*-catenin signaling underlies a wide range of pathologies in humans, including familial adenomatous polyposis (FAP), bone disease, and cancer [21]. Wnt/*β*-catenin signaling can be regulated by a degradation complex containing APC, Axin, glycogen synthase kinase 3*β* (GSK3*β*), and casein kinase I (CKI) [22]. The tumor suppressor APC is most commonly mutated and downregulated in many types of cancers, including lung adenocarcinoma, EBV-associated gastric carcinoma, nasopharyngeal cancer, and colorectal cancer [17, 23–26]. And downregulation of APC contributes to tumor progression and development. For instance, Lin et al. have demonstrated that miR-3607 was upregulated in lung cancer, and it promotes cell proliferation and tumorigenesis by directly suppressing APC expression in lung adenocarcinoma [24]. Another study has identified miR-494 that leads to alteration of APC/Wnt/*β*-catenin signaling and promotes cancer progression [25]. Herein, we demonstrated that APC expression is downregulated in CAEBV and NPC tissues compared to healthy controls and is negatively correlated with BART19-3p expression in CAEBV, which suggests that BART19-3p contributes to the pathogenesis of EBV-associated diseases.

## 5. Conclusion

In conclusion, our findings strongly revealed that elevated expression of EBV miR-BART19-3p promotes cell proliferation and suppresses apoptosis in EBV-associated diseases by suppressing the expression of APC. This study might be a very significant discovery and presented a new mechanism of cell proliferation induced by miR-BART19-3p, suggesting that miR-BART19-3p/APC may serve as a potential therapeutic target for the treatment of EBV-associated diseases.

## Data Availability

The data used to support the findings of this study are included within the article.

## Supplementary Materials

Supplementary Materials Supplemental Table 1: characteristics of CAEBV, EBV-HLH patients, and healthy controls. Supplemental Table 2: clinical and pathological characteristics of NPC patients. Supplemental Table 3: forward primers of EBV miRNAs used in this study.
