# Supplementary material for: The Oncogenic Role of miR-BART19-3p in Epstein-Barr Virus-Associated Diseases
Source: Biomed Res Int. 2020 Jul 2;2020:5217039. doi: 10.1155/2020/5217039 (PMC7354642; doi:10.1155/2020/5217039)
Supplement: Supplementary Materials — Supplemental Table 1: characteristics of CAEBV, EBV-HLH patients, and healthy controls. Supplemental Table 2: clinical and pathological characteristics of NPC patients. Supplemental Table 3: forward primers of EBV miRNAs used in this study. [file 5217039.f1.docx]

**Supplemental Table 1** Characteristics of CAEBV, EBV-HLH patients, and healthy controls.

| **Patients** | **CAEBV**  **(n=20)** | **EBV-HLH**  **(n=20)** | **Healthy Control**  **(n=10)** | ***P*-value** | | |
| --- | --- | --- | --- | --- | --- | --- |
|  |  |  |  | **CAEBV vs EBV-HLH** | **CAEBV vs Healthy Control** | **EBV-HLH vs Healthy Control** |
| Gender (Male/Female) | 12/8 | 9/11 | 5/5 | 0.342 | 0.602 | 0.796 |
| Age (Mean ± SD, years) | 4.6±2.5 | 5±3.3 | 4.9±2.8 | 0.635 | 0.769 | 0.896 |
| Whole blood EBV-DNA copies/μg DNA (Mean, Range) | 10^6.62^,  10^4.49^-10^7.59^ | 10^5.20^,  10^3.10^-10^6.32^ | <10^2.70^ | 0.086 |  |  |
| Plasma EBV DNA copies/ml (Mean, Range) | 10^3.88^,  10^2.70^-10^4.79^ | 10^3.01^,  10^2.70^-10^3.48^ | <10^2.70^ | 0.229 |  |  |

**Notes:** One-way ANOVA and Chi-square test were used for analysis. *P*-values <0.05 and EBV DNA copies <10^2.70^ were considered statistically significant or negative.

**Abbreviations:** CAEBV, chronic active EBV infection; EBV-HLH, EBV-associated hemophagocytic lymphohistiocytosis.

**Supplemental Table 2** Clinical and pathological characteristics of NPC patients

| **Patients** |  | **Characteristics** |
| --- | --- | --- |
| Gender | Male | 13 |
|  | Female | 1 |
| Age (Mean ± SD, years) |  | 46.7±16.2 |
| Pathological | undifferentiated NPC | 14 |
|  | differentiated NPC | 0 |
| EBV positive |  | 14 |
| Adjacent tissues |  | 4 |
| Mucosal chronic inflammation tissues |  | 3 |

**Supplemental Table 3** Forward primers of EBV miRNAs used in this study.

| EBV-miRNA | Forward primers | EBV-miRNA | Forward primers |
| --- | --- | --- | --- |
| BHRF1-1 | GCGTAACCTAATCAGCCCCG | BHRF1-2-3p | GCTCAGTCGTATCTTTTGCGGC |
| BHRF1-2-5p | GCGATACTGTTGCAGCAGATAGC | BHRF1-3 | GCGTAACGGGAAGTGTGTAAGCA |
| BART1-3p | GCGTATCACCGCTATCCACTATGTC | BART1-5p | TCTTAGTGGAAGTGACGTGCTGTG |
| BART2-3p | GCGAAGGAGCGATTTGGAGAA | BART2-5p | GCGTATTTTCTACATTCGCCCTT |
| BART3-3p | GTCGCACCACTTGTCACCAGG | BART3-5p | GCGACCGAGTGTTAGTGTTGTGCT |
| BART4-3p | GCACATCACATAGGCACCAGGA | BART4-5p | GACCTGATGCTGCTGGTGTGCT |
| BART5-3p | GCTGTAGTCCGCTGTTCACCA | BART5-5p | CAAGGTGAATATAGCTGCCCATCG |
| BART6-3p | GCGGGAATCGGACTAGCCTTA | BART6-5p | GGTAAGGTAGGTCCAATCCATAGG |
| BART7-3p | GCATCATAGTCCAGTGTCCAGGG | BART7-5p | GCCTGGACCTTGACTATGAAACA |
| BART8-3p | GCGTCACAATCTATGGGGTCGTA | BART8-5p | GCTCAGCGGTTTCCTAGATTGTAA |
| BART9-3p | GTAACACTTCATGGGTCCCGTAG | BART9-5p | GCTACTGGACCCTGAATTGGAAAC |
| BART10-3p | GCTACATAACCTTGGAGTTGGCTG | BART10-5p | GCTGCCACCTCTTTGGTTCTGTA |
| BART11-3p | GCTTCACGCACACCAGGCTA | BART11-5p | GTCAGACAGTTTGGTGCTCTAGTTG |
| BART12 | TCCTGTGGTGTTTGGTGTGGTT | BART13-3P | GGTAGATGTAACTTGCCAGGGACG |
| BART13-5p | GAACTGGCTCGTGGCTCGTA | BART14-3p | GCGTAAATGCTGCTGTAGTAGGGAT |
| BART14-5p | TACCCTACGCTGCCGATTTACA | BART15 | GCGGTCAGTGGTTTTGTTTCCTT |
| BART16 | GCGTTAGATAGAGTGGGTGTGTGCT | BART17-3p | TGTATGCCTGGTGTCCCCTTAGT |
| BART17-5p | TAAGAGGACGCAGGCATACAAG | BART18-3P | GTATCGGTAGTTTGGGCTTCGTC |
| BART18-5P | GCGTCCAGTTCGCACTTCCTAT | BART19-3p | TTTTGTTTGCTTGGGAATGCT |
| BART19-5p | ACATTCCCCGCAAACAAGACA | BART20-3p | GCATGAAGACACAGCCTGTTACC |
| BART20-5p | GGTAGCAGGCAAGTCTTCATTCC | BART21-3p | GCTAGTTGTGCCCACTGGTGTTT |
| BART21-5p | GCGTCACAACTGAAGGCAACTAAC | BART22 | GCGTTCAAAGTCGTGGTCTAGTAGT |
